# Solitary splenic metastasis secondary to endometrial adenocarcinoma: A case report

**DOI:** 10.1016/j.ijscr.2025.111752

**Published:** 2025-07-29

**Authors:** Ghena Alhadwah, Nahar Ismaiel, Jaafar Shater, Ali Daoud, Maen Haidar

**Affiliations:** a Department of Surgery, Tishreen University Hospital, Latakia, Syria; b Department of Pathology, Tishreen University Hospital, Latakia, Syria; c Faculty of Medicine, Tishreen University, Latakia, Syria; d Cancer Research Center, Tishreen University, Latakia, Syria

**Keywords:** Endometrial adenocarcinoma, Solitary splenic metastasis, Splenectomy, Hysterectomy, Case report

## Abstract

**Introduction:**

Endometrial adenocarcinoma is the most common gynecological malignancy and it usually metastasizes to different places. However, a solitary metastatic lesion to the spleen from endometrial carcinoma is a rare occurrence. Herein, we report a case of Solitary splenic metastasis secondary to endometrial adenocarcinoma.

**Presentation:**

A 64-year-old female presented to the emergency room complaining of severe constipation, abdominal pain, nausea, fever, fatigue, general weakness, and weight loss. Clinical exam showed signs of bowel obstruction and abdominal guarding. US followed by a CT scan showed a large pelvic mass and a hypo-intense lesion in the lower pole of the spleen and both were removed surgically. Pathological examination confirmed the diagnosis of a solitary splenic metastasis secondary to endometrial adenocarcinoma.

**Discussion:**

This case is the 20th case of its kind reported in the medical literature of a solitary splenic metastatic lesion originating from endometrial adenocarcinoma. Additionally, almost all of the cases published in the medical literature, to our knowledge, were cases of symptomatic endometrial adenocarcinoma which was treated accordingly until remission was achieved, then the disease relapsed as a metastatic lesion in the spleen which makes our case unique as the patient first presented with splenomegaly-related symptoms.

**Conclusion:**

The successful diagnosis and management of this rare presentation underscore the significance of a collaborative and multidisciplinary approach in the treatment of these patients.

## Highlights

• Endometrial adenocarcinoma is the most common gynecological cancer.
• Solitary metastatic lesion to the spleen from endometrial carcinoma is rare.
• The reason for the low frequency of metastases is unclear.
• US and CT both provide high accuracy, sensitivity and specificity in diagnosis.
• Our case was unique as the first symptoms that the patient experienced were those caused by splenomegaly.

## 1 Introduction

Endometrial cancer is considered the most common gynecological cancer in developed countries [[1], [2], [3]]. This condition can produce symptoms at an early stage. Thus, it is usually diagnosed in an early stage and has a good prognosis [2,3]. However, some patients can present with advanced primary disease or a recurrence of said disease [2]. Endometrial cancer most commonly metastasizes to the regional lymph nodes, ovaries, liver, lungs, bones, pleura, brain, bowel, and adrenal glands [1]. The spleen is a rare location for metastasis from solid tumors and the most common causes of these metastatic lesions are breast, lung, colorectal, ovarian cancers, nasopharyngeal carcinoma and melanomas [4,5]. Isolated metastasis to the spleen from endometrial carcinoma however is extremely rare [4,5], and to our knowledge, only 19 cases of solitary splenic metastasis of this common form of cancer are reported in the medical literature.

Herein, we present a rare case of endometrial adenocarcinoma accompanied by a solitary metastatic lesion in the spleen. This case has been reported in line with the SCARE criteria [6].

## 2 Presentation

A 64-year-old female presented to the emergency room complaining of severe constipation. Accompanied by abdominal pain, nausea, mild fever (38C), fatigue, general weakness, and weight loss of (15 kg over 3 months). Upon further interrogation, she noted that the constipation started 10 days ago and that she was able to pass gas throughout this period.

The patient had a past surgical history of appendectomy and cholecystectomy. No other significant medical, family, drug history, or allergies were noted. Finally, the patient is a heavy smoker (30 packs/year).

Clinical examination showed generalized abdominal pain, tenderness, and abdominal guarding which was most pronounced below the umbilicus. Laboratory studies showed HGB: 10.6 (g/dl), WBC: 20,600 (cells/ml), Gran: 65 %, MCV: 59.6(fl), CRP: 202 (mg/l), Urea: 55 (mg/dl), Total Bilirubin: 0.42 (mg/dl). Other laboratories were within the normal range.

**Fig. 1:**
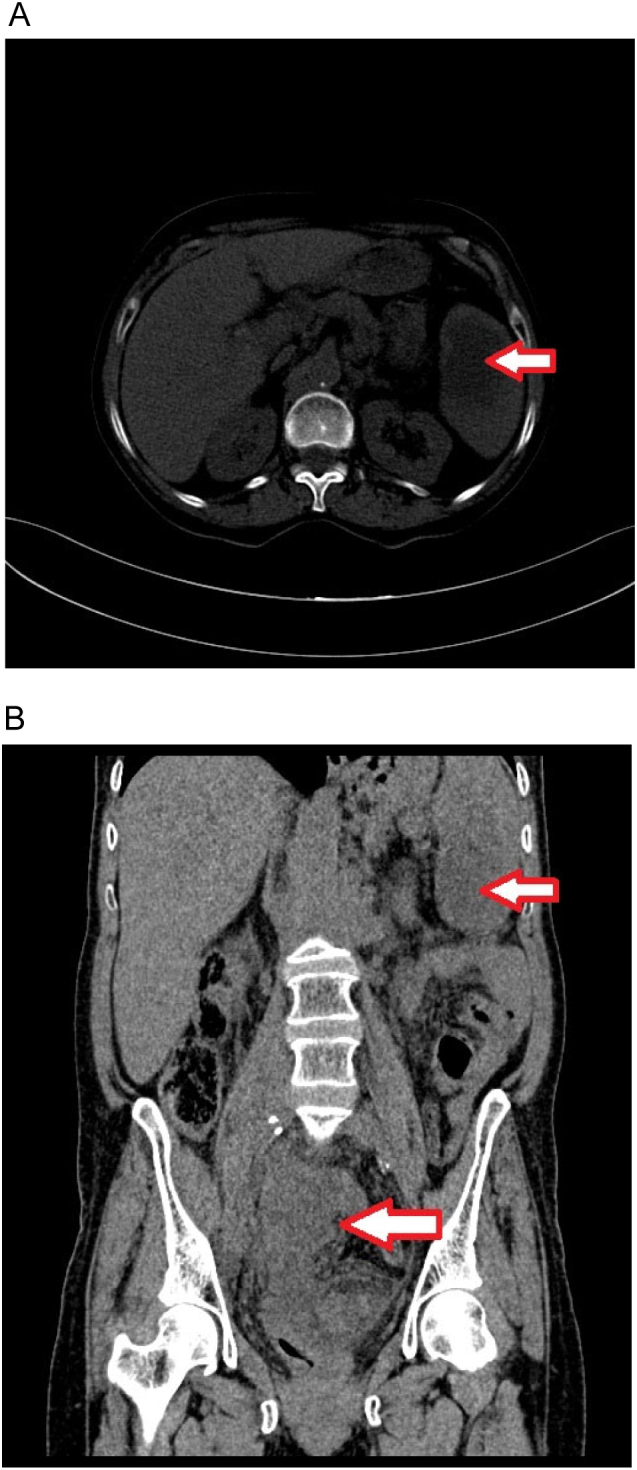
A. CT scan, axial view. The arrow shows a hypo-dense splenic mass. B. CT scan, coronal view. Arrows show a large uterine mass and a hypo-dense splenic mass.

An abdominal X-ray was ordered and it showed several differential air-fluid levels. The team suspected a bowel obstruction thus an abdominal ultrasound scan was done to further examine the abdomen and it showed an enlarged spleen with heterogeneous echotexture and a hypoechoic circular area (4 × 3 cm) in the lower splenic pole. However, said scan was unable to determine the cause of the aforementioned signs of obstruction. Therefore, a CT scan was done (Fig. 1) and it showed a large pelvic mass arising from the uterus (measuring 12.5 × 7 × 7 cm) and infiltrating the right adnexa which suggested the presence of endometrial carcinoma. Additionally, a hypo-intense lesion measuring 4 × 6 cm was found in the lower pole of the spleen which was most likely a metastatic lesion.

The medical team decided that the best course of action was an exploratory laparotomy which was to be performed by a multidisciplinary team including both general surgeons and gynecologists.

Under general anesthesia, a midline incision extending from the Xiphoid process to the symphysis pubis was done and sufficient dissection was undertaken to give the surgeons good access to the abdominal cavity and the pelvic organs.

Exploring the abdomen, an enlarged necrotic spleen was found with a mass in the lower pole. The splenic ligaments were tied and divided. This was followed by tying and dividing the splenic artery and vein. Finally, the spleen was removed and sent to pathology.

Exploring the pelvis, a large necrotic mass arising from the uterus was found. The mass extended to the right adnexa and formed adhesions with adjacent intestinal loops (explaining the obstruction signs and symptoms).

The intestinal adhesions were surgically released, the broad ligament was isolated and divided bilaterally using monopolar electrocautery, the cervix was divided, the remaining stump was sutured in a running fashion and the uterus was removed. Finally, the right and left adnexa (including the patient's ovaries) were resected and specimens were sent to the pathology department.

The surgical field was examined thoroughly and hemostasis was achieved using electrocautery. An intra-operative urology consult was requested to examine the bladder and no injuries were detected. Finally, the wound was closed in multiple layers. We note that due to intraoperative blood loss, two units of blood were transfused to the patient intraoperatively to maintain sufficient blood volume.

Post-operatively, the patient recovered well and her symptoms subsided.

Gross examination of the specimens revealed an enlarged deformed uterus measuring 4x6x8cm with a large mass infiltrating the serosa and right adnexa (12.5 × 7 × 7 cm) in addition to that the spleen was found to be enlarged with mass located in the lower pole measuring 4x6cm.

**Fig. 2:**
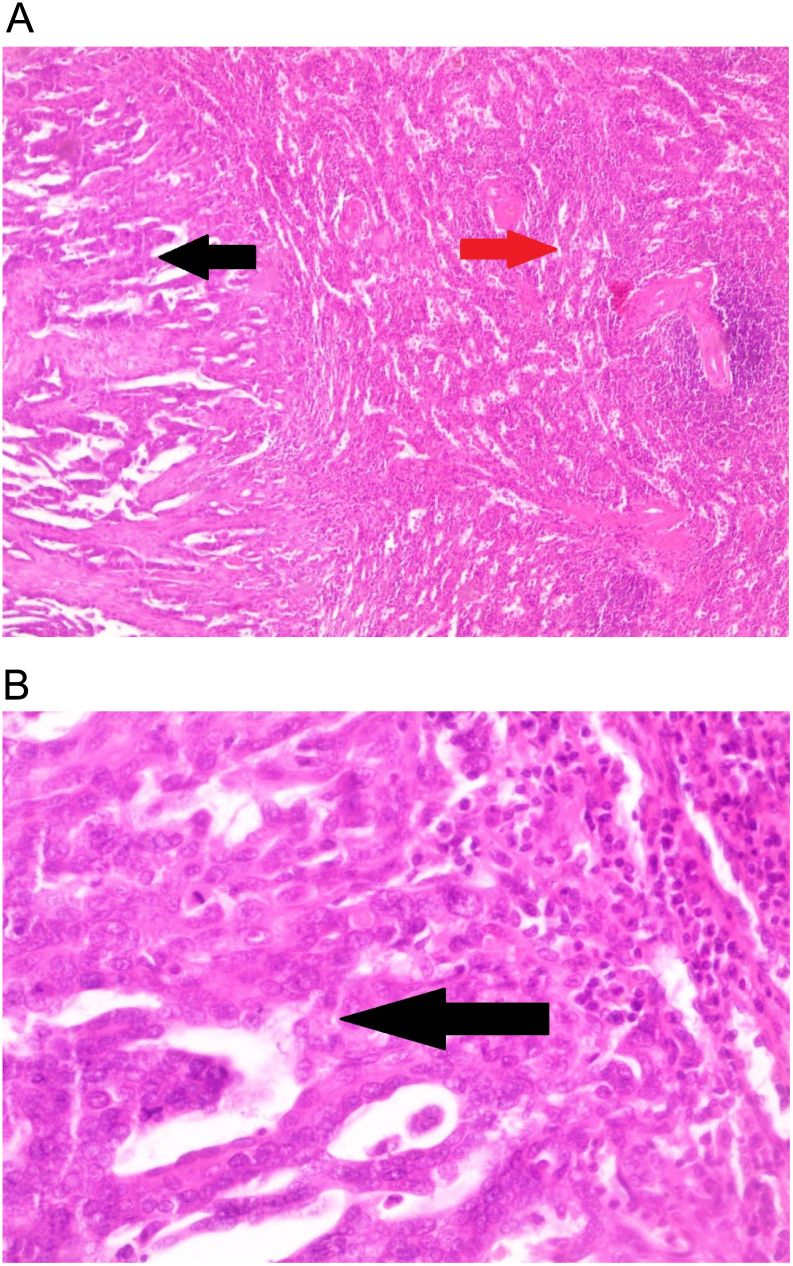
A. Spleen specimen (H&E 40×). The red arrow shows normal splenic tissue; the black arrow shows infiltrating endometrial adenocarcinoma (atypical glandular formations consisting of anaplastic epithelial cells). B. Splenic specimen (H&E 400×). The arrow shows anaplastic epithelial cells infiltrating the normal splenic tissue.

**Fig. 3:**
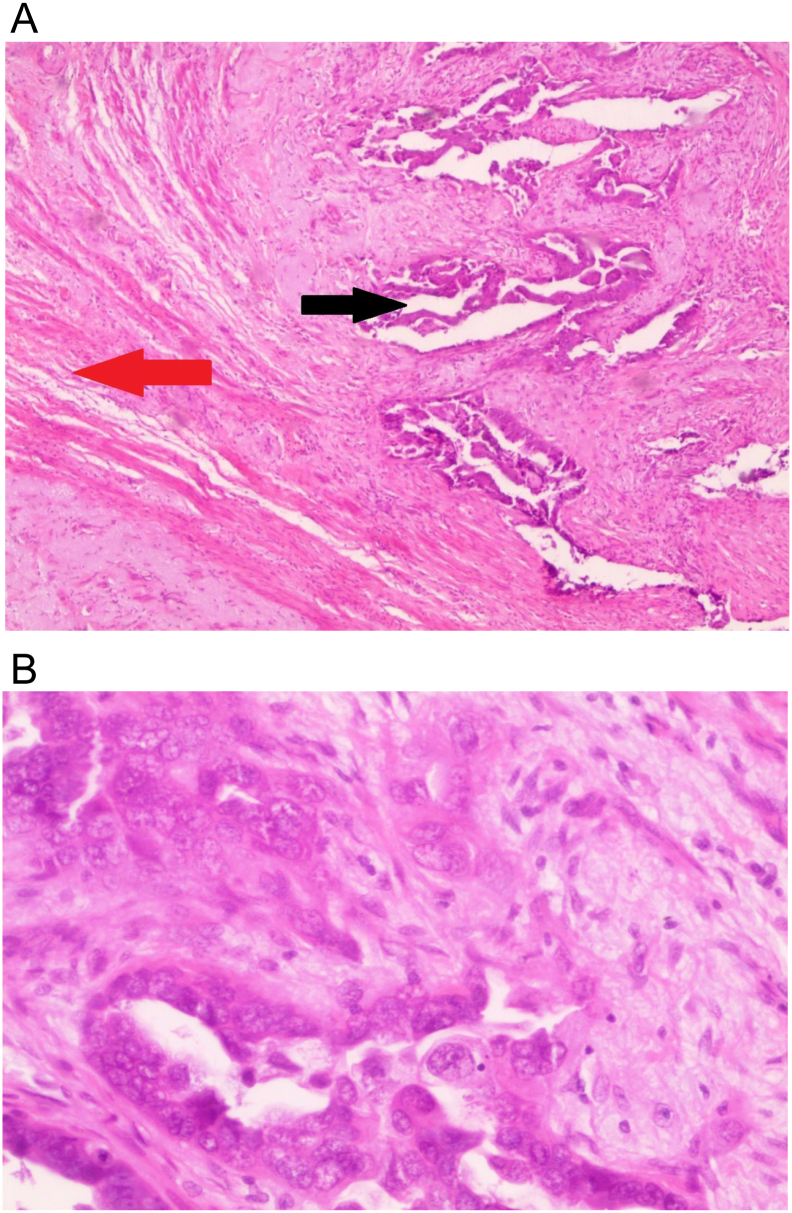
A. Uterine specimen (H&E 40×). The red arrow shows normal myometrium; the black arrow shows atypical glandular formations consisting of anaplastic epithelial cells which is consistent with endometrial adenocarcinoma. B. Uterine specimen (H&E 400×) anaplastic epithelial cells with enlarged nuclei and irregular mitotic figures.

Microscopic examination of the uterine specimen (Fig. 3) revealed atypical glandular formations consisting of anaplastic epithelial cells which infiltrate the uterine stroma. The splenic specimen (Fig. 2) showed atypical glandular formations similar to those found in the uterine specimen. Thus the final diagnosis of a poorly differentiated endometrial adenocarcinoma with splenic metastasis was made.

The patient was referred to the oncology team and she was put on a treatment plan consisting of three doses of Carboplatin (450 mg) and Paclitaxel (260 mg) with 21-day-intervals between each dose. She remains stable.

## 3 Discussion

Endometrial adenocarcinoma is a common gynecological cancer in developed countries [[1], [2], [3]]. There are four main mechanisms by which this cancer metastasizes; direct extension, trans-peritoneal spread, hematogenous, and lymphogenous routes. And the most common sites where the regional lymph nodes, ovaries, liver, lungs, bones, pleura, brain, bowel, and adrenal glands [1,2,7].

The reason for the low frequency of metastases is unclear. However, there are several theories that may explain this phenomenon as mentioned by stojanovic et al. and Teng et al.; such as the constant blood flow to the spleen, rhythmic contraction of the spleen, the sharp angle of the splenic artery branching, the lack of a lymphatic system, and the splenic microenvironment and special anatomy [5,8].

Almost 50 % of splenic metastases arise from the female genital tract with the most common origin being ovarian malignancies, followed by endometrial, cervical, and finally, tubal carcinomas [4]. Other origins include melanomas and breast carcinomas [1].

Solitary metastatic lesions to the spleen are a rare occurrence [4,8]. They usually arise from endometrial carcinoma and metastasize via the hematogenous route [1,2,5,8].

Most solitary metastatic lesions in the spleen are detected incidentally using different radiological tests like ultrasound, CT, or MRI and are usually asymptomatic [1,4,8].

When these lesions manifest clinically, they usually present with fatigue, weight loss, fever, abdominal pain, splenomegaly, anemia, thrombocytopenia. And sometimes they may present acutely as a splenic rupture [4,5].

On rare occasions splenic metastasis may be the first manifestation of solid cancers especially gynecological tumors [4].

We searched the related literature extensively and to our knowledge, only 19 cases of solitary splenic metastasis arising from endometrial carcinoma were reported in the medical literature which makes this case the 20th case of its kind reported in the literature. Additionally, almost all of the cases published in the medical literature, to our knowledge, were cases of symptomatic endometrial carcinoma which was treated accordingly until remission is achieved, then the disease relapsed as a metastatic lesion in the spleen. Our case however was unique in this perspective as the first symptoms that the patient experienced were those caused by splenomegaly and the uterine tumour was discovered incidentally via CT as was mentioned in the presentation.

Several imaging modalities can aid in diagnosing these lesions including ultrasound, CT, and MRI scans [4,8].

As MRI scans are expensive and not always available. The main comparison remains between US and CT scans as they both provide high accuracy, sensitivity and specificity. However, the micronodular pattern of splenic involvement found in US scans can give false positive findings [4].

The aforementioned diagnostic tools can be very helpful in diagnosing splenic metastasis. Nevertheless, the golden standard for diagnosing these lesions is the gross and microscopic pathological examination as it provides the definitive diagnosis [5].

The main form of treatment given to these patients is a splenectomy as it alleviates the discomfort of splenomegaly and protects the patient from complications like splenic rupture and splenic vein thrombosis. in addition, it also decreases the chance of further spreading of the cancer and reduces the tumour burden before adjuvant therapy is administered [2,4,8]. This operation should subsequently be followed by chemotherapy to reduce the chances of dissemination [1].

In our case, much emphasis was placed on multi-disciplinary treatment as the patient needed to have her reproductive organs as well as her spleen resected. Due to that, a team of general surgeons and gynecologists performed her operation.

## 4 Conclusion

Solitary splenic metastasis from endometrial carcinoma is a rare manifestation of a common condition. Herein we present a rare case of solitary splenic metastasis secondary to endometrial carcinoma which is the 20th case of such kind reported in the medical literature. The golden standard for diagnosing this condition is pathological examination and the best treatment protocol is splenectomy followed by adjuvant chemotherapy. Emphasis should be placed on the multidisciplinary approach to treatment especially if the discovery of the original tumour was due to the metastatic lesion. Thus maximizing treatment efficacy and patient safety and achieving the best possible outcome.

## Informed consent

Written informed consent was obtained from the patient for publication of this case report and accompanying images. A copy of the written consent is available for review by the Editor-in-Chief of this journal on request.

## Ethical approval

Given the nature of the article, a case report, no ethical approval was required.

## Guarantor

Prof. Maen Haidar.

## Research registration number

Not needed.

## Provenance and peer review

Not commissioned, externally peer-reviewed.

## Funding

No funding was required.

## Author contribution

All authors contributed to this manuscript.

Ghena Alhadwah: Writing - original draft, reviewing, and editing.

Nahar Ismaiel: Writing - original draft, reviewing, and editing.

Jaafar Shater: reviewing, and editing.

Ali Daoud: Reviewing and editing.

Maen Haidar: Supervision; final reviewing and editing

## Conflict of interest statement

The authors declare no conflict of interest.
